# Changes in self-related cognitive processes and depressive symptom severity following a single, moderate dose of psilocybin in depressed patients

**DOI:** 10.1098/rstb.2025.0168

**Published:** 2026-11-10

**Authors:** Lydia Belinger, Flora Moujaes, Robin von Rotz, Nathalie M. Rieser, Xaver Funk, Klemens Egger, Lena Meinhold, Maike Renkert, Philipp Stämpfli, Marcus Herdener, Franz X. Vollenweider, Katrin H. Preller

**Affiliations:** ^1^ Department of Adult Psychiatry and Psychotherapy, Psychiatric University Hospital Zurich, Zürich, Switzerland; ^2^ Magnetic Resonance Center, Psychiatric University Hospital Zurich, Zürich, Switzerland; ^3^ Department of Child and Adolescent Psychiatry, Psychiatric University Hospital Zurich, Zürich, Switzerland; ^4^ Leiden University Institute of Psychology, Leiden, The Netherlands; ^5^ Neuroscience Center Zurich, Zürich, Switzerland; ^6^ University Children's Hospital Zürich Center for MR Research, Zürich, Switzerland; ^7^ Zurich Center for Neuroeconomics, University of Zurich, Zürich, Switzerland

**Keywords:** psychedelic-assisted therapy, psilocybin, depression, self-referential processing, self-concept

## Abstract

Psychedelic-assisted therapy, particularly with psilocybin, is increasingly proposed as a treatment for major depressive disorder, yet the neural and psychological mechanisms underlying its effects remain under investigation. In a randomized, double-blind, placebo-controlled clinical trial, individuals diagnosed with major depressive disorder received a single, moderate dose of psilocybin (0.215 mg kg^−1^) or placebo, with the same psychological support. We analysed *N* = 37 individuals with major depressive disorder undergoing assessments of depressive symptoms (Beck's depression inventory and Montgomery–Åsberg depression rating scale), self-concept (Frankfurt self-concept scale) and neural markers of self-referential processing using an fMRI-based task. Compared with placebo, the psilocybin group exhibited significant reductions in depressive symptoms and a significant increase in positive self-concept two weeks after substance administration. Changes in self-concept were significantly correlated with decreased depressive scores. Neuroimaging revealed psilocybin-associated modulation of caudate activation during externally oriented attention after two weeks, contrasting with placebo-induced caudate changes during self-referential processing. These findings extend prior evidence linking psychedelics to changes in self-referential processing and self-concept and demonstrate that such changes occur concurrently with reductions in depressive symptoms. Future research should aim to replicate and extend these results and refine therapy protocols targeting self-processing and self-concept in psychedelic-assisted therapy in major depressive disorder.

This article is part of the discussion meeting issue ‘From being to identity: analysis and synthesis of the self’.

## 1. Introduction

The sense of self is a fundamental organizing principle of human consciousness, encompassing how individuals experience themselves as embodied, continuous and distinct from others. It shapes perception, emotion, cognition and social interaction, and disturbances in self-experience are implicated in a wide range of psychiatric disorders. Despite its centrality, the self remains a challenging construct to investigate experimentally, as many of its core features are implicit, stable over time, and difficult to manipulate in controlled settings. Identifying experimental approaches that can reliably induce marked alterations in self-experience is therefore crucial for advancing both basic and clinical research on the self.

Classic psychedelics such as psilocybin have emerged as particularly promising tools in this context. Through their action on the serotonin 2A (5-HT_2A_) receptor, psychedelics are capable of inducing profound yet transient alterations in consciousness, including marked changes in perception, mood, cognition and the sense of self [1,2]. Psychedelics are uniquely positioned to probe multiple levels of the self, as they affect immediate experiential modes of being as well as higher-order self-representations related to identity. As such, they provide a valuable opportunity to study the structure, flexibility and integration of self-experience and its neural underpinnings.

Early work demonstrated that psychedelics can acutely alter both bodily and cognitive dimensions of self-experience, including body representation, body boundaries, body awareness, body ownership, body location, autobiographical self and thought ownership [3]. These effects are thought to be mediated primarily by 5-HT_2A_ receptor stimulation [1,4]. More recent studies have replicated and extended these findings. For example, Preller *et al.* [4] showed that self-experience in the context of self- and other-initiated social interactions in healthy participants was acutely altered by LSD. These effects were blocked by the serotonin 2A receptor antagonist ketanserin, indicating a central role of 5-HT_2A_ receptor activation. Alterations in self-experience were associated with reduced differentiation between self and others in the LSD group and with decreased differential activation in cortical midline structures [4]—regions involved in self-modelling [5]. Furthermore, LSD increased the perceived self-relevance and meaningfulness of external stimuli [6]. Using a verbal self-monitoring task combined with EEG, Smigielski *et al.* [7] demonstrated that psilocybin modulated self–other processing and self–other distinction in healthy participants, with these effects correlating with subjective substance experiences such as unity and changed meaning. Psilocybin has also been shown to influence tactile deviancy processing and the integration of self- and body-related stimuli, pointing to additional sensory and bodily dimensions of self-experience affected by psychedelics [8]. These experimentally observed acute alterations of self-experience are reflected in subjective psychedelic experiences, including mystical-type experiences and phenomena such as ‘ego dissolution’—the perceived loss of self-boundaries and of the distinction between self and the external world [9]. Importantly, such experiences have been linked to therapeutic outcomes in depression [10–12], as well as in anxiety and substance use disorders [13–15].

Correspondingly, psychedelics have gained increasing attention as potential therapeutic agents. Psilocybin, in particular, has shown promise in the treatment of depression [11,16–18], as well as other psychiatric disorders, including anxiety [12,14] and substance use disorders [15,19–21]. Current research suggests that a single, moderate dose of psilocybin coupled with psychological support can lead to a significant reduction in depressive symptoms compared with placebo [16,18,22]. In light of the urgent need for faster-acting and more effective antidepressant treatments [23], these findings point to a compelling avenue for the development of novel therapeutic strategies. However, the mechanisms underlying the therapeutic effects of psilocybin remain incompletely understood. Identifying the psychological and neurobiological mechanisms through which psilocybin exerts its effects may improve the treatment efficacy of psychedelic-assisted therapy by targeting specific mechanisms that enhance treatment response.

One set of candidate mechanisms relevant to both depression and self-experience involves self-concept and self-referential information processing. Self-concept refers to the beliefs and evaluations that individuals hold about themselves, whereas self-referential processing describes the cognitive mechanisms by which information is interpreted in relation to oneself. Disturbances in self-concept are a central feature of depression and a strong predictor of its onset and severity [24]. Individuals with depression often exhibit negative self-schemas, such as internalized beliefs of worthlessness, and they preferentially process incoming information in a manner that reinforces these beliefs. Compared with non-depressed individuals, they show a tendency towards negatively biased self-referential processing, which contributes to a self-perpetuating cycle that maintains depressive symptomatology [25,26]. These processes may therefore represent important targets for therapeutic interventions aimed at alleviating depressive symptoms.

Given the central role of self-concept and self-referential processing in depression, and the capacity of psychedelics to transiently alter self-related experience, these substances may offer a novel means of addressing key features of depressive pathology. Within this context, the present study investigates how post-acute changes in self-concept and self-referential processing are associated with therapeutic outcomes following a single, moderate dose of psilocybin compared with placebo in patients with major depressive disorder (MDD). Self-referential cognitive processes were assessed using neuroimaging, and self-concept was measured using a self-report instrument, both up until two weeks after substance administration. By relating these measures to clinical outcomes, this study aims to elucidate potential mechanisms through which psychedelic-assisted therapy exerts its effects and to identify factors that may enhance treatment response, thereby contributing to a broader understanding of how changes across multiple levels of the self relate to psychopathology and recovery.

Our results show that psilocybin not only decreases depression severity scores, but it also increases associated positive self-concept and influences brain activation in the caudate during self-referential versus externally directed attention processing.

## 2. Methods

### (a) Study design and ethical considerations

Data for the present analyses were collected as part of the study by von Rotz *et al.* [18], which was conducted at the Psychiatric University Hospital in Zurich, Switzerland, between 11 April 2019 and 12 October 2021. A detailed description of study procedures and the primary endpoint is presented in [18]. In brief, the primary endpoint analyses showed that depressive symptoms were significantly reduced for at least two weeks after a single, moderate dose of psilocybin compared with placebo. The present manuscript builds on this prior work by focusing on depressive symptom reduction in relation to changes in self-concept and self-referential processing.

The study was conducted in a randomized, double-blind, placebo-controlled, parallel group design. Participants received either a single, moderate dose of psilocybin or a placebo, in combination with the same psychological counselling. Randomization was performed at the third visit using a computerized 1:1 allocation sequence without further stratification by the resident pharmacy.

The study comprised seven in-person visits: one screening visit (between −42 and −5 days relative to substance administration), two preparation visits (−3 days (+/−2 days) and −1 day relative to substance administration), one substance administration visit and three integration visits (+2 days, +8 days and +14 days relative to substance administration). A study timeline can be found in figure S1 in the supplementary material. All visits took place in a living room setting. At the substance administration visit, participants received either a single oral dose of synthetic psilocybin (0.215 mg kg^–1^; Compass Pathway Ltd.) or placebo (100% mannitol). Capsules were identical in size, weight, colour and shape. The psilocybin dosage was selected to correspond to a moderate psychoactive dose [27,28]. Following substance administration, participants were encouraged to focus inward and fully engage with the psychedelic experience. A standardized music playlist was played through headphones or speakers. A trained therapist was present throughout the substance administration visit to provide support as needed. All participants, regardless of group allocation, received six psychological counselling sessions adding up to 11 hours throughout the study. Details on the therapeutic framework are described in [18].

### (b) Participants and recruitment

The recruitment of participants took place through online advertisements, flyers, local and national newspapers and referrals from practising physicians. Individuals who met the following inclusion criteria were eligible for participation: Montgomery–Åsberg Depression Rating Scale (MADRS) score of 10–40 points, age between 20–60 years, right-handedness, no comorbidities, German language proficiency at level C2 and willingness to abstain from using psychotropic substances for the entire duration of the study. Psychiatric medication such as antidepressants had to be discontinued under psychiatric supervision at least two weeks or five half-lives before the day of substance administration. Ethnicity and sex of participants were determined by self-report. A complete list of inclusion and exclusion criteria can be found in table S1 in the supplementary material.

A total of 37 participants with MDD who completed all three MRI sessions were included in the statistical analyses, representing a subsample of the entire clinical cohort described by von Rotz *et al.* [18]. Figure S2 in the supplementary material presents a detailed consort flow diagram illustrating the study sample selection process. Demographic information on the study sample is listed in table 1. Prior psychedelic use was tested as a potential confound, but showed no significant main or interaction effects on any outcome measure (see supplementary material *prior psychedelic use*).

**Table 1.** Demographics for *N* = 37 patient participants. Age, sex and ethnicity of patients were determined by self-report. Verbal IQ was evaluated using the multiple-choice word test (MWT-B). Medication usage was documented during the medical screening prior to discontinuation. Prescription medications encompass all pharmaceuticals requiring a prescription, excluding antidepressants, which are reported separately. BDI = Beck's depression inventory, MADRS = Montgomery–Åsberg depression rating scale at baseline (−3 days relative to substance administration). Psychedelic use encompasses psilocybin and LSD. Owing to rounding, percentages may not sum up to 100%. The two groups did not significantly differ from each other in age or verbal IQ (two-sample *t*-test: *p*-corrected > 0.05).

	psilocybin (*n*= 19)	placebo (*n*= 18)
variable	M (SD/%)	M (SD/%)
age	37.3 (10.7)	33.7 (9.1)
verbal iq	117 (13.2)	113 (12.9)
sex		
female	9 (47.4%)	5 (27.8%)
male	10 (52.6%)	13 (72.2%)
ethnicity		
Caucasian	19 (100%)	16 (88.9%)
Arab	0 (0%)	1 (5.6%)
Black	0 (0%)	1 (5.6%)
BDI	26 (7.1)	24.3 (9.1)
MADRS	24.1 (4.4)	23.7 (6.9)
prior antidepressants		
no. of participants with 0	15 (78.9%)	13 (72.2%)
no. of participants with 1	3 (15.8%)	4 (22.2%)
no. of participants with > 1	1 (5.3%)	1(5.6%)
prior prescription medication		
no. of participants with 0	16 (84.2%)	11 (61.1%)
no. of participants with 1	2 (10.5%)	4 (22.2%)
no. of participants with > 1	1 (5.3%)	3 (16.7%)
prior psychedelic use	4 (21.1%)	8 (44.4%)

### (c) Depression severity

Depression severity was assessed at each of the seven study visits using both clinician-rated and self-report instruments.

MADRS is a structured 10-item interview conducted by a clinician, with each item scored from 0 to 6 (total score 0–60) [29]. Severity categories are: 0–6 = no symptoms, 7–19 = mild depression, 20–34 = moderate depression and 35–60 = severe depression [30].

The revised version of the Beck's depression inventory (BDI), BDI-II, is a 21-item self-report questionnaire with items rated from 0–3, for a total score of 0–63 [31]. Severity categories are: 0–9 = minimal depression, 10–18 = mild depression, 19–29 = moderate depression and 30–63 = severe depression.

For statistical analyses, scores from time points –3 days, –1 day, +2 days, and +14 days relative to the substance administration day were used. To verify initial equivalence between the psilocybin and placebo groups, Welch's two-sample *t*-tests were conducted on baseline BDI and MADRS scores (–3 days). Changes in BDI and MADRS scores across time were evaluated using mixed-design repeated-measures ANOVA frameworks with time point as the within-subject factor (–3 days, –1 day, +2 days, +14 days; e.g. the change in depression scores from –3 to +14 days) and group as the between-subject factor (psilocybin versus placebo). For significant time point x group interactions, we performed pairwise comparisons across time points within each group, with Bonferroni correction applied. Analyses were performed using RStudio v. 4.4.3 [32], and statistical significance was defined as *p* < 0.05 (two-tailed).

### (d) Frankfurt self-concept scale

The Frankfurt self-concept scale (FSCS) [33] comprises 10 one-dimensional scales with a total of 78 items, designed to assess various facets of self-concept. It was assessed at –3 and +14 days relative to the substance administration day. The FSCS subscales include: general performance (10 items), general problem solving (10 items), confidence in behaviour and decision-making (6 items), general self-esteem (10 items), sensitivity and mood (6 items), steadfastness towards groups and significant others (12 items), communication and interaction skills (6 items), appreciation by others (6 items), influence by others (6 items) and feelings and relationships towards others (6 items). Each scale employs a 1–6 Likert self-report format. The evaluation is done separately for each scale using the summed Likert scoring. The different scales show positive intercorrelations with each other. Across all subscales, higher sum scores denote a positive self-concept, while lower scores indicate a negative self-concept [34,35]. A detailed description of the different subscales can be found in the supplementary material *FSCS: subscales*.

The same statistical analyses as for depression scores were conducted for each FSCS subscale individually: mixed-design repeated-measures ANOVA (time point = within-subject factor; group = between-subject factor) and subsequent pairwise comparisons across time points within each group (Bonferroni corrected). Independent two-sample *t*-tests examined potential baseline differences between the two groups.

### (e) Correlation analysis

As exploratory analyses, we conducted Pearson's product–moment correlation to assess the relationships among changes in depression (BDI and MADRS) and self-concept measures (FSCS subscale scores) (i) across all patients and (ii) separately within each treatment group (psilocybin versus placebo). We focused on data showing significant interactions from the mixed repeated-measures ANOVA as well as subsequent *post-hoc* comparisons. Owing to the exploratory nature of these analyses, we did not implement a correction for multiple comparisons.

### (f) fMRI task and imaging protocol

#### (i) fMRI task design and data acquisition

The self-referential processing paradigm was adapted from Davey *et al.* [36]. It consisted of two conditions: self-referential processing and an external attention condition. Participants completed 10 blocks per condition, which were presented in an intermixed block design separated by a rest–fixation period, for a total of 20 blocks per participant. Each block consisted of five personality adjectives presented for 5 s each, during which participants indicated a yes/no response. In the self-referential processing condition they responded to the question ‘Does this word describe you?’ with a press on the left or right button on a magnetic resonance (MR)-compatible response pad. In the external attention condition, adjectives were rated for the question ‘Does this word have four or more vowels?’. The adjectives were drawn from a validated list [37], centred around the median likeableness rating and selected for meaningfulness [38] (e.g. skeptical, perfectionist and lucky). Word lists for both active conditions were matched for likeableness and vowel count and counterbalanced across patients. Each block (3-s instruction + five words × 5 s) was followed by a jittered rest–fixation period (mean of 6 s), during which participants were instructed to focus on a centrally presented crosshair.

Imaging data were acquired during three of the seven visits: –1, +2 and +14 days relative to the substance administration. Each MRI session lasted approximately 1 hour and included two anatomical scans (T1- and T2-weighted), one resting-state scan and two-task based scans, including the self-referential processing paradigm. A Philips Achieva 3.0T MRI scanner (Best) with a 32-channel head coil and multi-transmit parallel radio frequency transmission system was used to acquire magnetic resonance data. Functional images were acquired with a whole-brain gradient-echo planar imaging (EPI) sequence (2400 ms repetition time; 27 ms echo time; 3 mm slice thickness; 45 axial slices; without slice gap; 240 × 240 mm^2^ field-of-view; 3 × 3 mm^2^ in-plane resolution; 2.0 sensitivity-encoding reduction factor). For obtaining high-resolution anatomical images, a standard T1-weighted three-dimensional magnetization sequence with gradient echo was used (MPRAGE; 12 ms repetition time, 5.7 ms echo time, 0.7 mm slice thickness, without interslice gap, 0.7 × 0.7 × 0.7 mm^3^ voxel size).

#### (ii) Behavioural analysis

Behavioural analysis included a repeated-measures ANOVA with condition (self, external) and time point (–1 day, +2 days and +14 days) as within-subject factors and reaction time (RT) as the dependent variable. Follow-up paired-samples *t*-tests with Bonferroni correction to adjust for multiple comparisons were applied. To examine potential group effects, an additional mixed-design ANOVA was conducted including group (psilocybin versus placebo) as a between-subjects factor and condition and time point as within-subject factors.

#### (iii) Preprocessing and motion analysis

Image preprocessing and analyses were conducted in MATLAB (R2022b) [39] using Statistical Parametric Mapping version 12 (SPM12) [40,41]. Preprocessing included slice-time correction, realignment, spatial normalization to the standard Montreal Neurological Institute (MNI) EPI template, and spatial smoothing with an 8 mm FWHM Gaussian kernel. Two patients were excluded from further analysis based on excessive head movement, defined as displacement equal to or exceeding the size of one voxel  3 mm^–1^. Framewise displacement analysis did not reveal any significant differences in motion between groups, time points or significant interaction effects (supplementary material, figure S6).

#### (iv) fMRI first and second level analysis

Onset times for self-referential processing, external attention condition and rest–fixation were extracted and aligned to the scanner's trigger signalling. Condition-specific timing files were generated for each patient. Preprocessed fMRI data and onset regressors were further analysed with a general linear model implementation using the SPM12 software package to create a model with the three main regressors plus six movement regressors, which were used for a subsequent second-level flexible factorial design in SPM12 with three factors: group (psilocybin versus placebo; between-subjects), condition (self, external; within-subjects) and time point (–1 day, +2 days, +14 days; within-subjects). Significant clusters for the contrast self > external over all time points were identified at *p-FWE* < 0.05 (cluster-level, based on an initial threshold of *p* < 0.001, uncorrected; supplementary material, figure S3 and table S2). For a total of seven significant brain clusters, we extracted their eigenvariates and tested them for group differences and changes in activity over time using a mixed-design repeated-measures ANOVA framework in RStudio v. 4.4.3 [32] (time point = within-subject factor; group = between-subject factor). We tested main effects of group, condition and time point as well as their interactions (including all two-way and three-way group × condition × time point interactions). Subsequently, we tested the significant three-way interactions with pairwise comparisons across time points within each group × condition combination (Bonferroni corrected). Furthermore, we correlated changes in significant brain clusters with significant changes in depressive symptoms and FSCS subscale scores.

All statistical tests used a significance level of *p* < 0.05 and were conducted in two-tailed fashion. Significant brain clusters were visualized in the MNI-space, using MRIcroGL [42].

## 3. Results

### (a) Depressive symptom reduction

BDI and MADRS baseline scores (–3 days) did not differ significantly between the psilocybin and placebo groups (BDI: *p* = 0.539; MADRS: *p* = 0.864). The mixed repeated-measures ANOVA revealed a significant group × time point interaction for both outcome measures (*p* < 0.0001). For both BDI and MADRS scores, subsequent Bonferroni-corrected pairwise comparisons revealed a significant reduction in depressive symptoms in the psilocybin group from –3 to +2 days (*p* < 0.0001; BDI: 95% CI [12.0 to 20.3], *d* = 1.90; MADRS: 95% CI [11.1 to 17.6], *d* = 2.13), –3 to +14 days (*p* < 0.0001; BDI: 95% CI [10.3 to 19.8], *d* = 1.52; MADRS: 95% CI [10.2 to 17.6], *d* = 1.82), –1 to +2 days (*p* < 0.0001; BDI: 95% CI [11.9 to 19.0], *d* = 2.10; MADRS: 95% CI [11.0 to 16.8], *d* = 2.33) and –1 to +14 days (*p* < 0.0001; BDI: 95% CI [10.2 to 18.5], *d* = 1.66; MADRS: 95% CI [9.8 to 17.2], *d* = 1.76) relative to the substance administration day. By contrast, the placebo group exhibited a significant decrease only in MADRS scores from –1 to +2 days (*p* < 0.05, 95% CI [1.3 to 7.2], *d* = 0.72) relative to the substance administration day. These findings indicate that changes in depression scores over time depended on treatment condition. Detailed results are illustrated in figure 1A.

**Figure 1.**
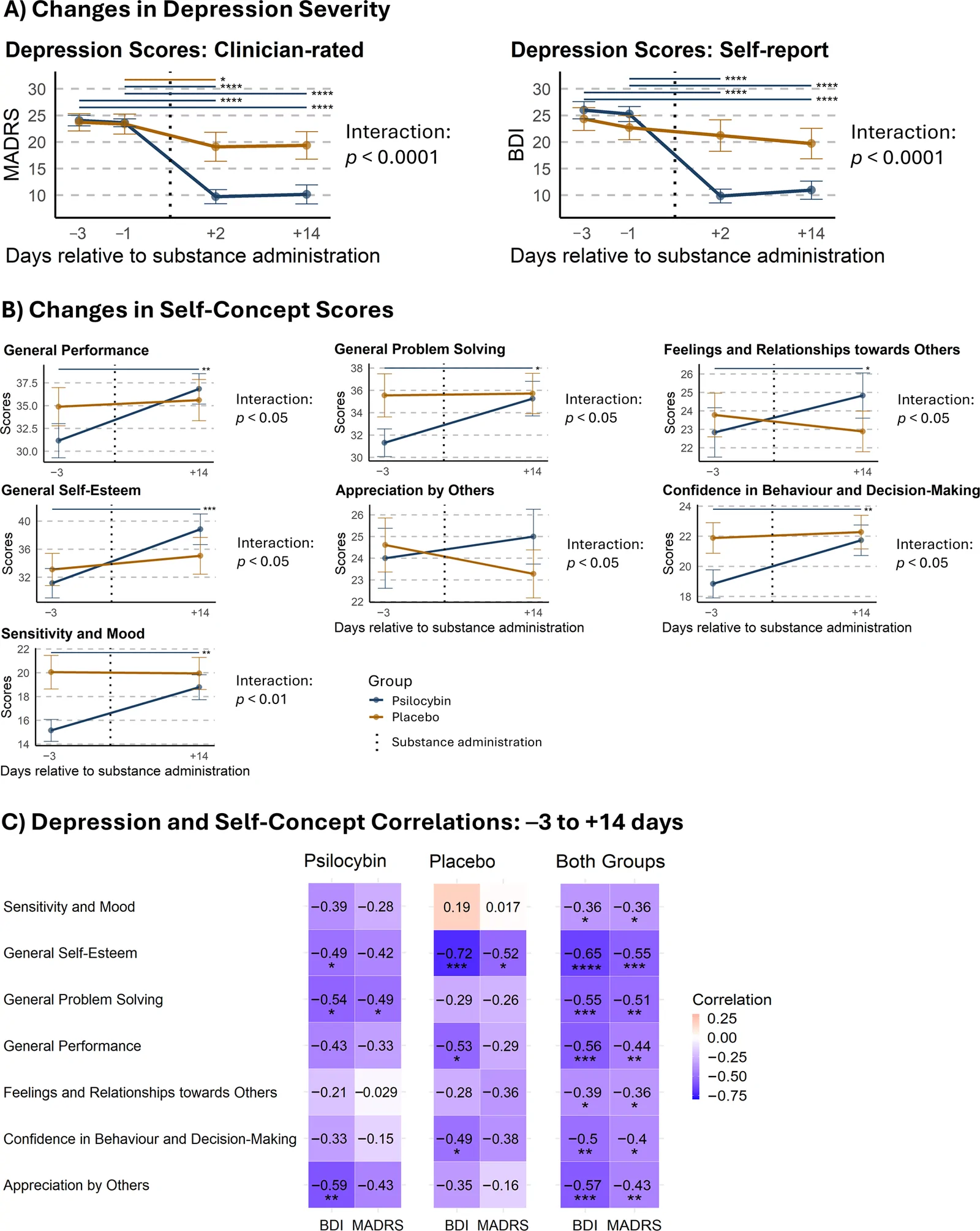
Changes in depression severity and self-concept scales. (A) Significant reduction in BDI and MADRS scores in the psilocybin group from –3 to +2 days, –3 to +14 days, –1 to +2 days and –1 to +14 days. Significant reduction in MADRS scores in the placebo group from –1 to +2 days. (B) Group x time point interactions for depicted FSCS subscales all reached significance. Significant increase for the psilocybin group in FSCS subscale scores from –3 to +14 days: improvements in general problem solving, feelings and relationships towards others, general performance, confidence in behaviour and decision-making, sensitivity and mood and general self-esteem. Appreciation by others did not reach significance in either group. (C) Correlations between changes in depression severity and FSCS subscale scores across both groups and for both groups individually. All correlations reached significance in the pooled analysis and some correlations reached significance in the individual substance groups (psilocybin: general self-esteem and BDI, general problem solving and BDI, general problem solving and MADRS, appreciation by others and BDI; placebo: general self-esteem and BDI, general self-esteem and MADRS, general performance and BDI, confidence in behaviour and decision-making and BDI. * = *p* < 0.05, ** = *p* < 0.01, *** = *p* < 0.001 and **** = *p* < 0.0001. *P*-values in panels (A) and (B) are Bonferroni-corrected, whereas correlation analyses in panel (C) were exploratory and therefore not corrected for multiple comparisons. Results in panels (A) and (B) are depicted as mean values with standard error bars.

### (b) Changes in self-concept domains

The mixed repeated-measures ANOVA conducted on the FSCS subscales revealed a significant group × time point interaction for seven scales: general performance, general problem solving, appreciation by others, feelings and relationships towards others, general self-esteem, confidence in behaviour and decision-making (all *p* < 0.05) and sensitivity and mood (*p* < 0.01). The remaining three subscales did not reach statistical significance (*p* > 0.05). Only within the psilocybin group, six subscales demonstrated a significant increase in scores from –3 to +14 days relative to the substance administration day, as indicated by Bonferroni-corrected pairwise comparisons: general performance (*p* < 0.01, 95% CI [–9.38 to –1.99], *d* = –0.74), general problem solving (*p* < 0.05, 95% CI [–6.87 to –1.02], *d* = –0.65), feelings and relationships towards others (*p* < 0.05, 95% CI [–3.71 to –0.29], *d* = –0.58), general self-esteem (*p* < 0.001, 95% CI [–11.6 to –3.75], *d* = –0.94), confidence in behaviour and decision-making (*p* < 0.01, 95% CI [–4.46 to –1.33], *d* = –0.89) and sensitivity and mood (*p* < 0.01, 95% CI [–5.65 to –1.61], *d* = –0.87). The subscale appreciation by others did not reach significance in either group (*p* > 0.05). We found significant baseline differences between groups for the confidence in behaviour and decision-making (*p* < 0.05) and sensitivity and mood (*p* < 0.01) subscales, with higher values in the placebo than in the psilocybin group. No significant baseline differences were observed for the remaining subscales. These baseline discrepancies should be taken into account when interpreting change scores. Overall, results suggest that psilocybin administration leads to increases in FSCS subscale scores two weeks later, indicating an improved self-concept in this group. The findings are depicted in figure 1B.

### (c) Associations between reduced depressive symptoms and increase in positive self-concept

The exploratory correlation analyses between changes in depression and FSCS subscale scores (–3 to +14 days) revealed negative correlations within both the psilocybin and placebo groups, as well as in the combined sample, with only negligible exceptions (figure 1C). In the pooled analyses, every depression × FSCS subscale correlation reached statistical significance (*r* ranging from –0.36 to –0.65, all *p* < 0.05). When examining each group separately, the psilocybin sample showed significant negative correlations between changes in depression scores and three of the FSCS subscales: general self-esteem and BDI (*r* = –0.49, *p* < 0.05, 95% CI [–0.77 to –0.05]), general problem solving and both BDI (*r* = –0.54, *p* < 0.05, 95% CI [–0.8 to –0.12]) and MADRS (*r* = –0.49, *p* < 0.05, 95% CI [–0.77 to –0.04]) and appreciation by others and BDI (*r* = –0.59, *p* < 0.01, 95% CI [–0.82 to –0.18]) In the placebo group, significant negative correlations were found between changes in general self-esteem and both BDI (*r* = –0.72, *p* < 0.001, 95% CI [–0.89 to –0.39]) and MADRS (*r* = –0.52, *p* < 0.05, 95% CI [–0.79 to –0.07], general performance and BDI (*r* = –0.53, *p* < 0.05, 95% CI [–0.8 to –0.08]), and confidence in behaviour and decision-making and BDI (*r* = –0.49, *p* < 0.05, 95% CI [–0.78 to –0.04]).

The negative correlations indicate that a decrease in depression symptoms was associated with an increase in FSCS subscale scores, within both psilocybin and placebo groups separately, as well as across both groups. Figure 1C displays a detailed correlation matrix. Scatter plots can be found in figures S4 and S5 in the supplementary material.

### (d) Changes in brain activation during self-referential processing

For behavioural analysis, repeated-measures ANOVA revealed a significant main effect of time point (*p* < 0.001) and a significant main effect of condition (*p* > 0.001) on RT, with no significant interaction (*p* > 0.05). Participants responded significantly faster in the self-referential condition than in the external attention condition across all time points (–1 day: mean difference = 0.28 s, *p* < 0.001; +2 days: mean difference = 0.24 s, *p* < 0.01, +14 days: mean difference = 0.25 s, *p* < 0.01). Irrespective of condition, RTs were significantly lower at +2 days (*p* < 0.01) and +14 days (*p* < 0.001) compared with –1 day, with no significant difference between +2 days and +14 days (*p* > 0.05; mean RT: –1 day = 2.02 ± 0.29 s, +2 days = 1.90 ± 0.31 s, +14 days = 1.86 ± 0.28). The mixed-design ANOVA including group as a between-subjects factor revealed no main effect of group and no interactions involving group (all *p* > 0.05), but the same main effects of time point and condition (both *p* < 0.001). Differences in RTs between task conditions likely reflect differences in task demands and the reduction in RTs over time is consistent with task familiarization.

On a neural level, baseline group comparisons for both self-referential and external attention conditions showed no significant differences in brain activation between the psilocybin and placebo groups. Within the self > external contrast across time points, seven clusters reached significance (cluster-level *p-FWE* < 0.05), with peak-level brain activations in the superior frontal gyrus, pyramids, cingulate gyrus, middle temporal gyrus (MTG) and caudate. Visualization and further information on significant brain clusters are reported in figure S3 and table S2 in the supplementary material. The subsequent mixed repeated-measures ANOVA revealed a significant three-way group × condition × time point interaction (*p* < 0.001) for the caudate (cluster-level, *p* < 0.05, kE = 97, peak-level T = 5.49, MNI coordinates [12, 11, 14], based on an initial threshold of *p* < 0.001, uncorrected; shown in figure 2A). Subsequent pairwise analyses revealed divergent effects by group and condition. In the psilocybin group, activation during the external attention-related condition increased significantly in the caudate from –1 to +14 days (*p* < 0.01, 95% CI (–0.56 to –0.2), *d* = –1) and +2 to +14 days (*p* < 0.05, 95% CI (–0.45 to –0.05), *d* = –0.61) relative to the substance administration day. By contrast, in the placebo group, the caudate activation increased significantly during the self-referential processing condition from –1 to +2 days (*p* < 0.05, 95% CI (–0.53 to –0.07), *d* = –0.66) and –1 to +14 days (*p* < 0.01, 95% CI (–0.6 to –0.19), *d* = –0.95) relative to the substance administration day. All other comparisons were not significant. These findings suggest distinct changes in caudate-brain activation during the two task conditions, depending on the substance received. Results are presented in figure 2B.

**Figure 2.**
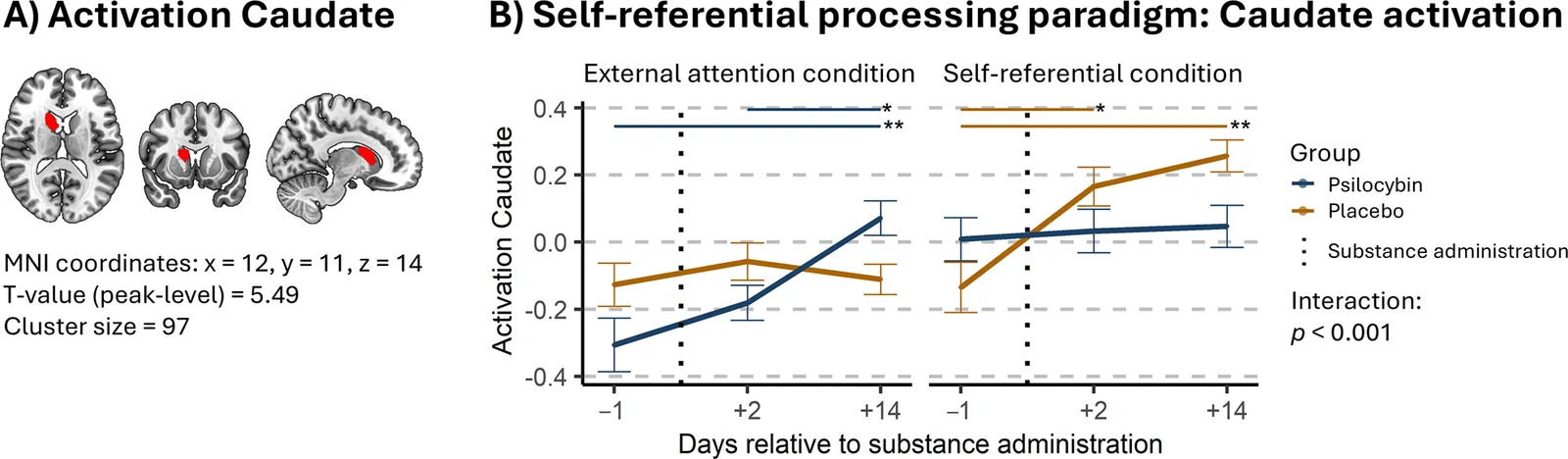
Brain activation during the self-referential processing paradigm. (A) Caudate activation extracted from the flexible-factorial design using the contrast self > external over all time points. (B) Caudate activation by MR-condition and substance group. Significant increase in caudate activation during the external attention condition for the psilocybin group: –1 to +14 days and +2 to +14 days. Significant increase in caudate activation during the self-referential condition for the placebo group: –1 to +2 days and –1 to +14 days. (*p* < 0.05). * = *p* < 0.05 and ** = *p* < 0.01. All *p*-values are Bonferroni corrected. Results in panel (B) are depicted as mean values with standard error bars.

Correlation analyses between changes in caudate activation, changes in depressive symptoms and changes in FSCS subscale scores did not reach significance.

## 4. Discussion

### (a) The relevance of self-concept in psychedelic-assisted therapy

Consistent with the primary endpoint findings reported by von Rotz *et al.* [18], participants in the psilocybin group of the present subsample exhibited a significant reduction in depressive symptoms that was sustained up to 14 days post-administration, compared with the placebo group. This effect was evident across both self-reported and clinician-rated measures of depression. Beyond symptom reduction, several domains of self-concept—as assessed by the FSCS subscales—showed significant improvements in the psilocybin group from baseline to 14 days post administration, whereas no comparable changes were observed in the placebo group. This suggests that psilocybin was associated not only with reductions in depressive symptoms, but also with improvements in depression-related self-concept.

Improvements were observed across multiple domains of self-concept, including general performance, general problem solving, sensitivity and mood, feelings and relationships towards others, general self-esteem and confidence in behaviour and decision-making. Baseline differences between groups for the confidence in behaviour and decision-making and sensitivity and mood subscales should be taken into account when interpreting change scores; nevertheless, significant increases over time were observed exclusively in the psilocybin group. Taken together, these findings position self-concept as a central psychological domain associated with therapeutic change following psilocybin administration in MDD.

A negative self-concept is a well-established feature of depression. Prior work has demonstrated that patients with depression who show greater improvements in self-esteem following cognitive behavioural interventions also exhibit steeper reductions in depressive symptoms, whereas individuals with smaller improvements in self-esteem tend to show less symptom reduction. Importantly, while these results indicate that changes in self-esteem and depressive symptoms co-vary over time, no inferences regarding a causal relationship can be drawn [43].

We find the same pattern in our results: in the psilocybin group, reductions in depressive symptoms were accompanied by increases in positive self-concept across several FSCS domains. Changes in depression severity and self-concept were negatively correlated in both treatment groups and across the pooled sample. Improvements in self-concept may, at least in part, reflect reductions in depressive symptoms over time, possibly owing to general therapeutic effects, rather than substance-specific effects alone. Notably, although negative correlations were present in both treatment groups, significant increases in self-concept occurred only in the psilocybin group. This pattern suggests that psilocybin administration may be associated with positive changes in self-concept that co-occur with symptom improvement, potentially contributing to therapeutic efficacy in MDD. However, given the correlational nature of these analyses, further work is required to disentangle substance-specific effects from general therapeutic improvements and to clarify causal relationships.

Overall, these findings highlight self-concept as an important psychological domain in the treatment of MDD, as well as a potential target for psychedelic-assisted therapy. Future studies employing longitudinal and mechanistic designs will be necessary to determine whether changes in self-concept play a causal role in symptom improvement.

### (b) Neural correlates of self-referential processes

Another core characteristic of depression is heightened self-focus, characterized by persistent, repetitive and self-critical processing, often termed ruminative brooding [44,45]. Neuroimaging studies have implicated the medial prefrontal cortex and other cortical midline structures in these processes, revealing abnormally increased activity associated with self-referential processing [45]. Using the same fMRI self-referential processing paradigm employed in the present study, Davey *et al.* [36] found that in depression, self-referential processing engaged the same dynamic brain network observed in healthy individuals; however, the depressed group showed enhanced top-down regulation from the medial prefrontal cortex to the posterior cingulate cortex. This was expressed as a stronger negative (inhibitory) modulatory influence on effective connectivity between the two regions during self-referential processing. Furthermore, the strength of this inhibitory modulation was inversely associated with symptoms of poor concentration and inner tension, but not with total MADRS scores [36].

Our findings indicate that when examining self-referential processing in contrast to an external attention condition, brain activation in the caudate varied between conditions and substance groups. Specifically, in the psilocybin group, caudate activation during the external attention condition increased from pre-administration to 14 days post-administration, with a further significant increase between 2 and 14 days post-administration. By contrast, the placebo group showed increased caudate activation during the self-referential condition from pre-administration to 2 days and 14 days post-administration. Taken together, these findings indicate that changes in caudate activation over time differed between the psilocybin and placebo groups in a task-dependent manner, with increased engagement during external attention in the psilocybin group and during self-referential processing in the placebo group.

Contrary to expectations based on prior work, no significant changes were observed in medial prefrontal or posterior cingulate regions [36], even though these brain areas were engaged by the task (supplementary material, figure S3). This absence may reflect a combination of factors, including our limited sample size, the exploratory whole-brain approach or a genuine lack of involvement of these regions in psilocybin-driven changes. Further research with larger samples and targeted hypotheses will be required to clarify these possibilities.

While caudate involvement emerged from the data-driven, exploratory whole-brain analysis, the finding is consistent with prior literature implicating the caudate nucleus in depression across a range of cognitive, emotional and affective tasks [46]. A well-replicated finding is reduced caudate activation during reward processing in individuals with depression compared with healthy controls [47]. Research further indicates that self-compassion is associated with increased functional connectivity between the left caudate and several brain regions—including the bilateral posterior superior temporal sulcus/temporoparietal junction, the left MTG and the left middle occipital gyrus—during positive compared with negative self-referential processing across both depressed and healthy adolescents. Furthermore, enhanced connectivity between the left caudate and MTG is suggested as a potential neural mechanism underlying the association between greater self-compassion and lower suicide ideation [48].

Evidence regarding psychedelic effects on caudate activation remains limited; however, a recent pilot fMRI study in individuals with alcohol use disorder reported increased caudate activation and caudate–anterior cingulate connectivity following psilocybin administration. The authors suggest these changes may reflect altered top-down cognitive control and reduced emotional reactivity post-treatment [49].

In the present study, increased caudate activation during the external attention condition in the psilocybin group may reflect altered attentional engagement during task performance, potentially indicating a reduced dominance of self-referential processing relative to externally directed attention. By contrast, increased caudate activation during self-referential processing in the placebo group may reflect greater engagement with self-evaluative cognition over time, possibly related to psychological support or repeated task exposure. Importantly, changes in caudate activation were not significantly correlated with changes in depressive symptoms or self-concept measures, indicating that these neural findings should be interpreted as complementary rather than as direct mediators of therapeutic outcome.

Several limitations should be considered when interpreting these results. The self-referential processing condition may engage cognitive and emotional processes beyond self-reference *per se*, and the external attention condition may not be fully matched in terms of cognitive demands. These potential differences may in part be reflected in different RTs in the self-referential compared with external attention condition. Additionally, the observed group × condition × time point interaction indicates that neural changes over time depended on both task context and treatment group; together with the modest sample size, this complexity warrants cautious interpretation. Replication in larger samples will be essential to establish the robustness of these findings.

## 5. Future considerations and conclusion

Psilocybin-assisted therapy shows promise for the treatment of MDD, as reflected in sustained reductions in both self-reported and clinician-rated depressive symptoms. The present findings further indicate that psilocybin administration is associated with post-acute improvements across multiple domains of self-concept, which co-occur with reductions in symptom severity. These results highlight self-concept as a psychologically meaningful domain associated with therapeutic change and a potential target for psychedelic-assisted interventions.

At the neural level, the present findings indicate task-dependent changes in neural engagement over time that differed between treatment groups. Within this pattern, psilocybin was associated with increased caudate activation during the external attention condition of the fMRI task, whereas the placebo group showed increased caudate activation during self-referential processing, suggesting that non-pharmacological factors associated with the intervention may engage neural processes distinct from those observed following psilocybin.

The present study is limited by its sample size and by the correlational nature of the observed associations, which preclude causal inferences. Future research should employ larger samples and longitudinal designs enabling formal mediation analyses to clarify the temporal and causal relationships between changes in self-concept, neural processing and clinical outcomes. Additionally, formal assessments of expectancy and blinding integrity should be incorporated to address potential confounds.

Taken together, these findings support the view that psychedelic-assisted therapy may influence depression-related psychopathology by engaging multiple levels of the self, encompassing subjective self-concept, self-referential cognitive processing and their neural correlates. By providing an experimental context in which these dimensions of self-experience can be studied longitudinally, psychedelics may offer a valuable framework for advancing both the scientific understanding of the self and the development of more targeted interventions for depression.

## Supplementary Material



## Data Availability

The data used for analysis are available on github: https://github.com/lydia-belinger/Belinger-et-al.-2026-Philosophical-Transactions-B/blob/main/. Supplementary material is available online [51].

## Theme

One contribution of 17 to a discussion meeting issue ‘From being to identity: analysis and synthesis of the self’.
