# Supplementary material for: Changes in self-related cognitive processes and depressive symptom severity following a single, moderate dose of psilocybin in depressed patients
Source: Philos Trans R Soc Lond B Biol Sci. 2026 Nov 10;381(1962):20250168. doi: 10.1098/rstb.2025.0168 (PMC13750421; doi:10.1098/rstb.2025.0168)
Supplement: Supplementary file 1 [file rstb.2025.0168.zip › rstb.2025.0168/rstb20250168_si_001.pdf]

royalsocietypublishing.org/journal/rstb Phil. Trans. R. Soc. B 0000000

1 Supplement

The following supplemental material belongs to the paper *Changes in self-related cognitive processes and depressive symptom severity following a single, moderate dose of psilocybin in depressed patients* by Belinger et al., 2025.

(a) Inclusion and Exclusion Criteria

Table 1. Inclusion and exclusion criteria for study participation

**Inclusion criteria**
Capability of giving informed consent by a signature
Age range of 20-60 years
Right-handedness
DSM-IV diagnosis of mild or moderate major depressive episode without psychotic symptoms (based on clinical assessment and confirmed by SCID interview)
Montgomery-Åsberg Depression Rating Scale (MADRS) score between 10 and 40 points
Discontinuation of psychotropic medication (at least 2 weeks or five half-lives prior to substance administration visit)
Absence of past and acute suicidal ideation (judged clinically)
Good somatic health with no unstable medical conditions (as determined by medical history, physical examination, routine blood labs, electrocardiogram, urine analysis, and urine toxicology)
German or Swiss-German as first language and normal language comprehension
Willingness to refrain from drinking alcohol the day before testing days and during testing days, from drinking caffeinated drinks during the testing days, and from using psychoactive substances 2 weeks prior and during the course of the study
Availability of a family member or friend to pick them up and stay with them overnight after the substance administration visit
People of childbearing potential must be using an effective, established method of contraception for the entire study duration
**Exclusion criteria**
Lifetime history of bipolar disorder (I, II, not otherwise specified), schizophrenia, schizoaffective disorder, or psychosis not otherwise specified
Alcohol or other drug dependence (DSM-IV) or abuse in the three months prior to enrollment (except for caffeine or nicotine)
Comorbid Axis I anxiety disorders permitted if no current treatment is required
Family history of schizophrenia or schizoaffective dirorder, or bipolar disorder type I (first or second degree relatives)
Current psychotherapeutic or psychological treatment from third parties during the study duration
Pregnant or breastfeeding patients
Abnormal electrocardiogram
Any unstable somatic illness as determined by history or laboratory tests
BMI < 17 or > 35
Uncorrected hypo- or hyperthyroidism
Participants who are pregnant or breastfeeding
Magnetic resonance imaging contraindications
History of allergy, hypersensitivity, or adverse reactions to hallucinogens
Lifetime history of hallucinogen use on more than 10 occasions
New use or dose changes of concomitant medication without informing the investigators
Symptoms associated with an increased risk of adverse emotional and/or behavioral reactions to treatment intervention based on investigator's clinical evaluation
Participation in another study with drug intake within the prior 30 days or during the present study

(b) Study timeline

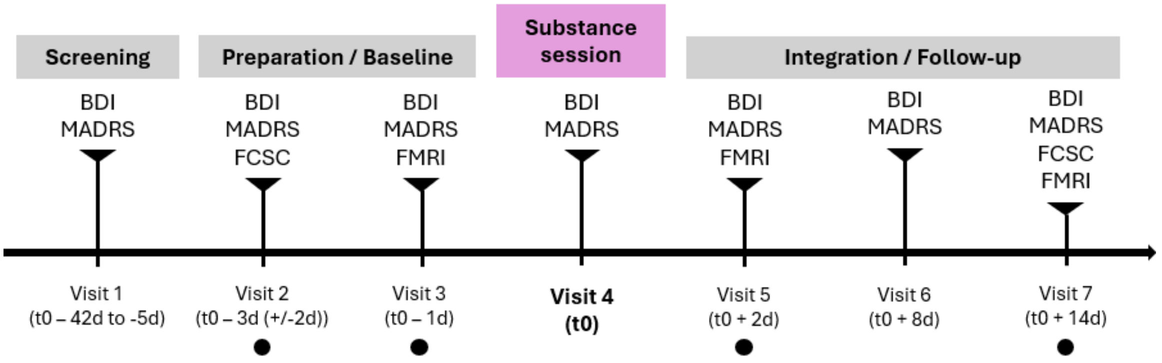

Figure 1. Study timeline with all in-person visits and measures included in the analyses (BDI, MADRS, FSCS, fMRI). The study comprised seven visits. Four timepoints were used for analyses, indicated by circles below the corresponding visits: Visit 2 (t0 - 3d (+/-2d)), Visit 3 (t0 - 1d), Visit 5 (t0 + 2d), and Visit 7 (t0 + 14d). BDI and MADRS used for analyses were collected at Visits 2, 3, 5, and 7; FSCS at Visits 2 and 7; and fMRI at Visits 3, 5, and 7.

(c) Consort Diagram

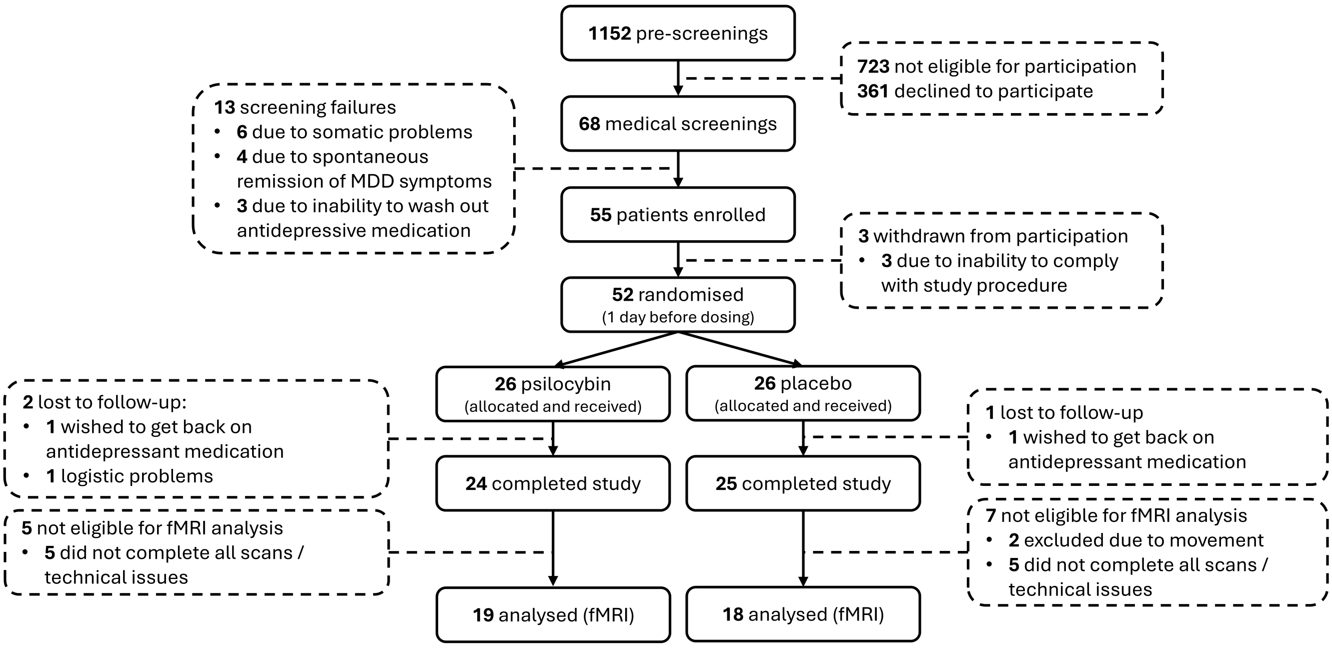

Figure 2. Consort Flow Diagram: Flow-chart depicting inclusion, randomization, and participation of participants throughout the study.

royalsocietypublishing.org/journal/rstb *Phil. Trans. R. Soc. B* 0000000

(d) Prior psychedelic use

To assess potential confounding of our outcomes by prior psychedelic use, we included prior use (yes/no) as a between-subject factor in the ANOVA models of depressive symptom scores (BDI and MADRS), FSCS scores, and fMRI caudate eigenvariates.

Inclusion of prior psychedelic use revealed no significant main effects or interactions with group, timepoint, or condition across outcome measures (BDI, MADRS, FSCS, fMRI caudate eigenvariates; all $p > 0.05$). Conclusions for depression scores and fMRI outcomes remained unchanged after inclusion of prior psychedelic use in the ANOVA models. In the FSCS analyses, some interaction effects were no longer statistically significant. Specifically, the group × timepoint interactions for the Sensitivity and Mood and Feelings and Relationships towards Others subscales remained significant ($p < 0.05$), whereas interactions for the other subscales were no longer statistically significant (General Performance, General Problem Solving, Confidence in Behaviour and Decision-Making, General Self-Esteem, and Appreciation by Others). Given the limited sample size, inclusion of prior psychedelic use reduced statistical power, likely contributing to this loss of significance.

(e) Frankfurt Self Concept Scale: Subscales

The FSCS subscales include: General Performance, General Problem Solving, Confidence in Behaviour and Decision-Making, General Self-Esteem, Sensitivity and Mood, Steadfastness towards Groups and Significant Others, Communication and Interaction Skills, Appreciation by Others, Irritability by Others, and Feelings and Relationships towards Others. For all subscales, items include general expressions, allowing respondents from diverse backgrounds to relate the items to their specific situations. The questionnaire was conducted in German.

General Performance: The General Performance subscale assessses individuals' perception in terms of cognitions, opinions, evaluations, feelings and behaviour related to their own performance with 10 items. The sum scores range from 10 to 60. Sum scores of $\geq$40 indicate a positive self-concept, while sum scores $\leq$30 reflect a negative self-concept.

General Problem Solving: The subscale of General Problem Solving uses 10 items to measure an individual's beliefs about their ability to independently address and overcome everyday problems and difficulties. The sum scores range from 10 to 60. Sum scores of $\geq$40 indicate a positive self-concept, while sum scores $\leq$30 reflect a negative self-concept.

Confidence in Behaviour and Decision-Making: The Confidence in Behaviour and Decision-Making subscale captures an individual's attitude towards their own sense of security in relation to their behaviour and decisions in everyday life with a total of 6 items. The sum scores range from 6 to 36. Sum scores of $\geq$24 indicate a positive self-concept, while sum scores $\leq$18 reflect a negative self-concept.

General Self-Esteem: The General Self-Esteem subscale refers to individual's overall attitudes towards themselves, including self-esteem and feelings of satisfaction or dissatisfaction with their own person. It includes a total of 10 items. The sum scores range from 10 to 60. Sum scores of $\geq$40 indicate a positive self-concept, while sum scores $\leq$30 reflect a negative self-concept.

Sensitivity and Mood: The Sensitivity and Mood subscale covers an individual's perception and emotions associated with their own sensibility, sensitivity, vulnerability and mood with 6 items. The sum scores range from 6 to 36. Sum scores of $\geq$24 indicate a positive self-concept, while sum scores $\leq$18 reflect a negative self-concept.

Steadfastness towards Groups and Significant Others: The Steadfastness towards Groups and Significant Others subscale encompasses 12 items to assess the individual's attitude towards situations and behaviour in various social contexts. It includes the perception of and emotions towards social confrontations with others (incl. authority figures) and the expression of one's own ideas and opinion. The sum scores range from 12 to 72. Sum scores of $\geq$48 indicate a positive self-concept, while sum scores $\leq$36 reflect a negative self-concept.

Communication and Interaction Skills: The subscale of Communication and Interaction Skills assesses the individual's attitude towards their ability and confidence in making contact and interacting with others with 6 items. The sum scores range from 6 to 36. Sum scores of $\geq$24 indicate a positive self-concept, while sum scores $\leq$18 reflect a negative self-concept.

Appreciation by Others: The Appreciation by Others subscale comprises 6 items to assess an individual's perception of how they are valued and regarded by their environment as well as the trust placed in them by their environment. The sum scores range from 6 to 36. Sum scores of $\geq$24 indicate a positive self-concept, while sum scores $\leq$18 reflect a negative self-concept.

Influence by Others: The Influence by Others subscale encompasses 6 items to cover an individual's attitude towards themselves in relation to the perceived influence of others on themselves (e.g. on their own perceptions, emotions and behaviour). The sum scores range from 6 to 36. Sum scores of $\geq$24 indicate a positive self-concept, while sum scores $\leq$18 reflect a negative self-concept.

Feelings and Relationships towards Others: The subscale of Feelings and Relationships towards Others covers an individual's attitude towards people in their environment in terms of their own cognition, opinions, thoughts, and emotions with a total of 6 items. The sum scores range from 6 to 36. Sum scores of $\geq$24 indicate a positive self-concept, while sum scores $\leq$18 reflect a negative self-concept.

(f) Self>external contrast across groups and timepoints: Significant brain clusters

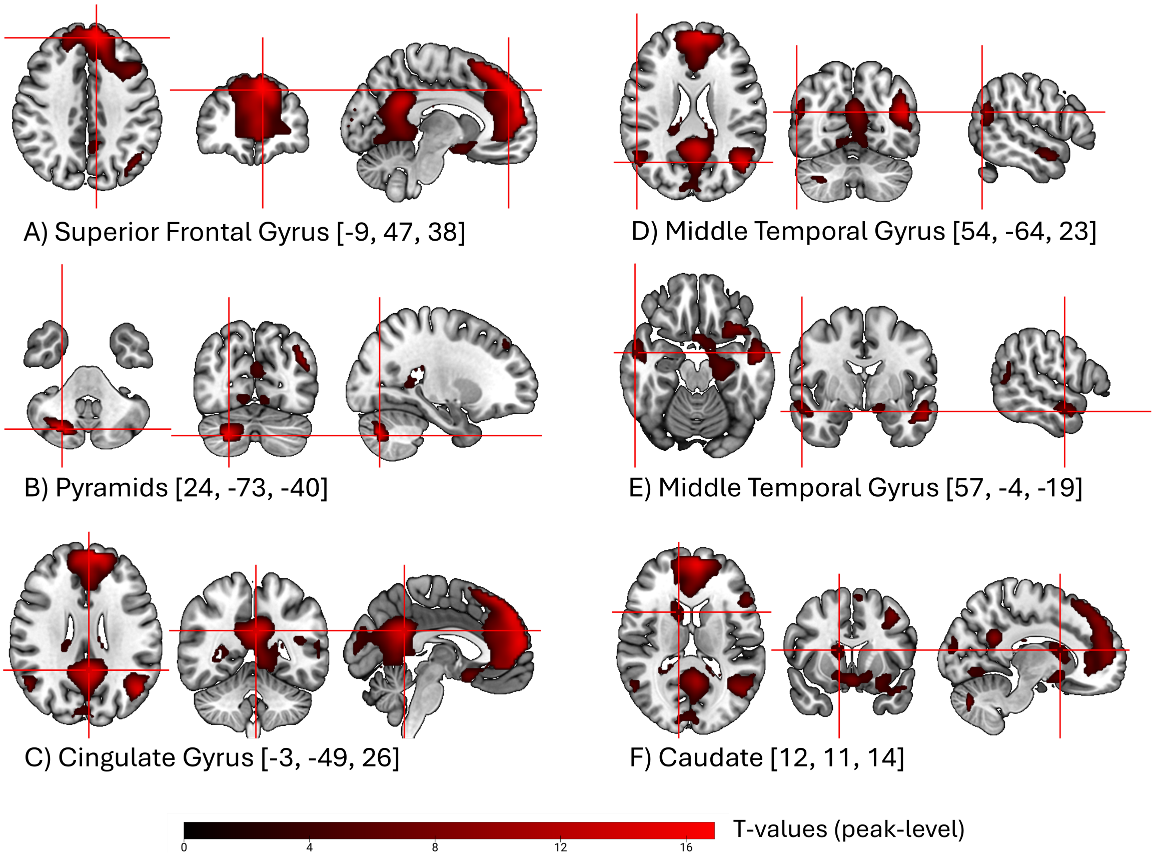

Figure 3. Significant brain clusters derived from the full flexible design for the contrast self > external over all timepoints at cluster-level. Crosshairs mark peak-level activations. Brain coordinates are put as MNI coordinates. *p-FWE*<0.05, cluster-level

Table 2. Significant brain clusters derived from the full flexible design for the contrast self > external over all timepoints (cluster-level $p < 0.05$)

**Brain region**	**MNI coordinates (peak-level)**	**Cluster size (kE)**	**T (peak-level)**
Superior Frontal Gyrus	x = -9, y = 47, z = 38	4548	16.96
Pyramids	x = 24, y = -73, z = -40	122	16.62
Cingulate Gyrus	x = -3, y = -49, z = 26	1516	14.30
Middle Temporal Gyrus	x = 54, y = -64, z = 23	95	9.72
Middle Temporal Gyrus	x = 57, y = -4, z = -19	93	7.11
Caudate	x = 12, y = 11, z = 14	97	5.49

(g) Correlation analyses

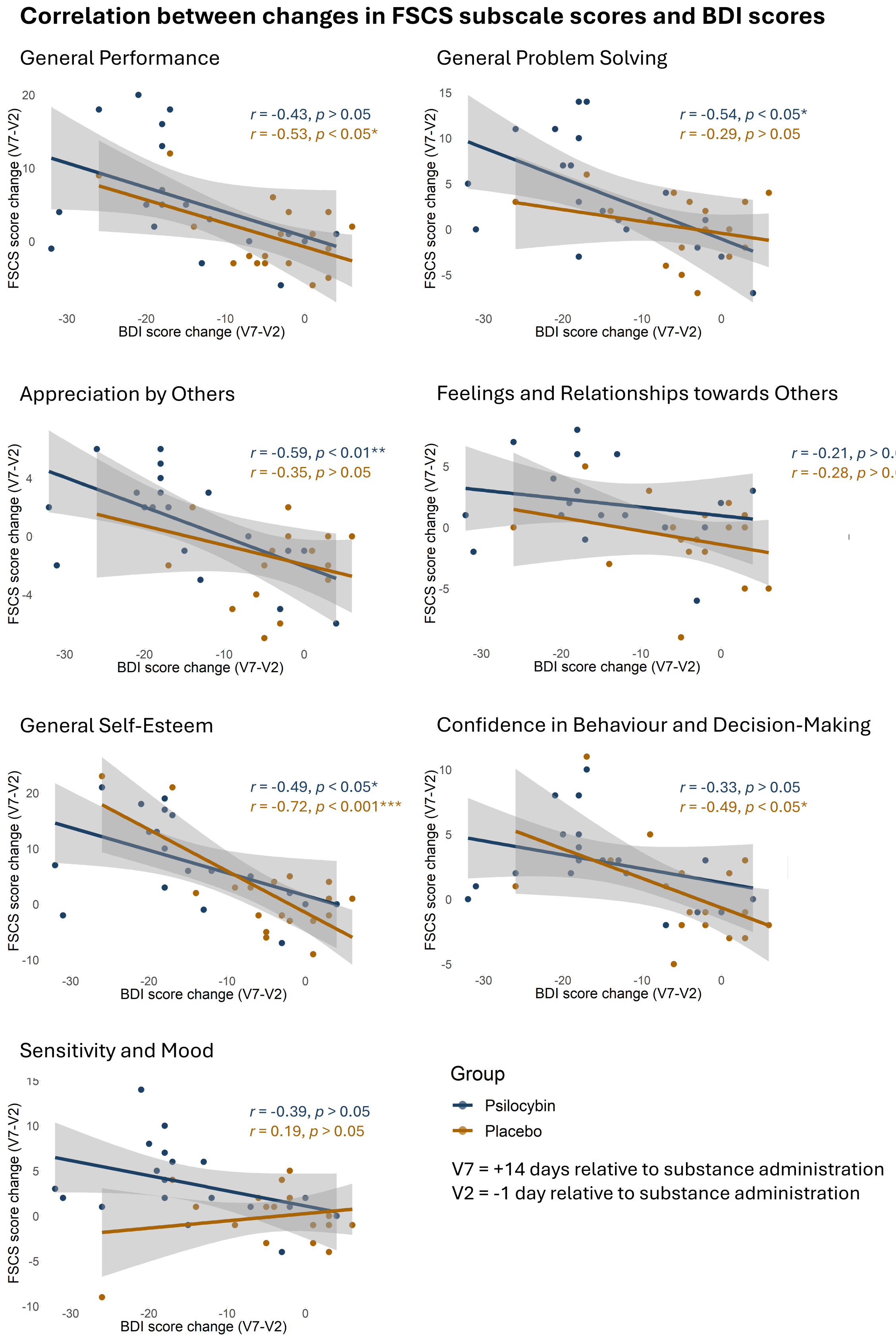

Figure 4. Pearson's product-moment correlation to assess the relationships between changes in Frankfurt Self Concept Scale (FSCS) subscale scores and Beck's Depression Inventory (BDI) scores, separately within the placebo and the psilocybin group. Differences from visit 7 (+14days) to visit 2 (-1 day) were used for correlation analyses.

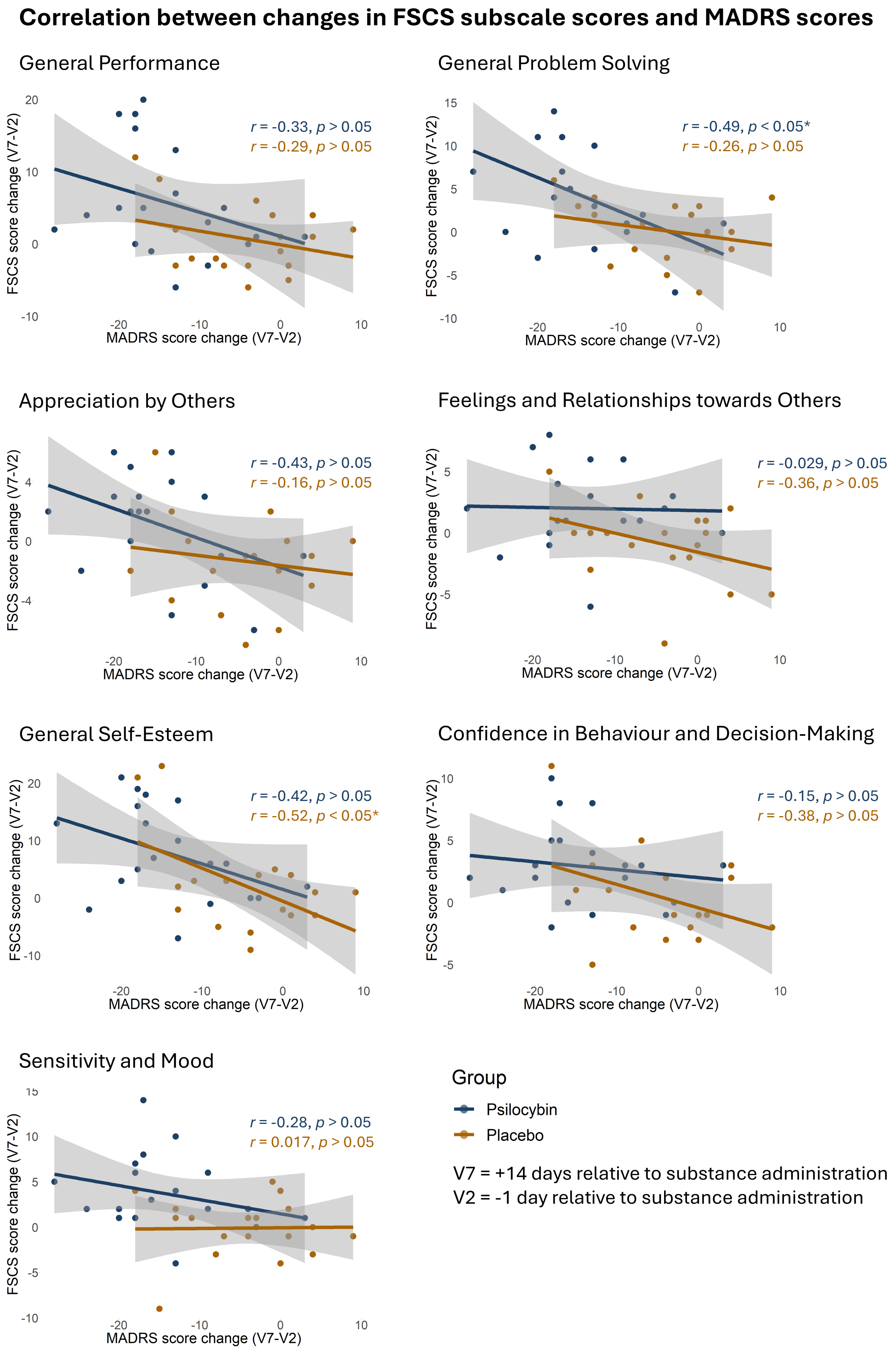

Figure 5. Pearson's product-moment correlation to assess the relationships between changes in Frankfurt Self Concept Scale (FSCS) subscale scores and Montgomery-Åsberg Rating Scale (MADRS) scores, separately within the placebo and the psilocybin group. Differences from visit 7 (+14days) to visit 2 (-1 day) were used for correlation analyses.

(h) Framewise Displacement

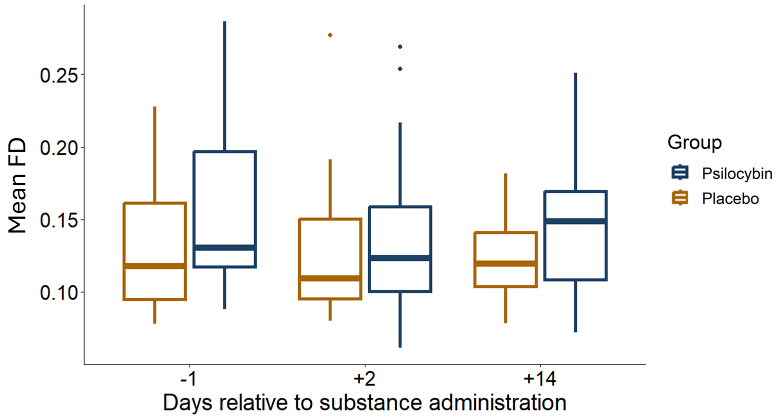

Figure 6. Framewise displacement (FD) analyses revealed no significant differences in FD between groups, timepoints or significant interaction effects, conducting a 2x3 mixed ANOVA.
